# A backwards glance at words: Using reversed-interior masked primes to test models of visual word identification

**DOI:** 10.1371/journal.pone.0189056

**Published:** 2017-12-15

**Authors:** Colin J. Davis, Stephen J. Lupker

**Affiliations:** 1 University of Bristol, Bristol, United Kingdom; 2 University of Western Ontario, London, Ontario, Canada; National Institutes of Health, UNITED STATES

## Abstract

The experiments reported here used “Reversed-Interior” (RI) primes (e.g., *cetupmor*-COMPUTER) in three different masked priming paradigms in order to test between different models of orthographic coding/visual word recognition. The results of Experiment 1, using a standard masked priming methodology, showed no evidence of priming from RI primes, in contrast to the predictions of the Bayesian Reader and LTRS models. By contrast, Experiment 2, using a sandwich priming methodology, showed significant priming from RI primes, in contrast to the predictions of open bigram models, which predict that there should be no orthographic similarity between these primes and their targets. Similar results were obtained in Experiment 3, using a masked prime same-different task. The results of all three experiments are most consistent with the predictions derived from simulations of the Spatial-coding model.

## Introduction

Successfully identifying a printed word requires a reader to encode the input stimulus, match this input code against stored lexical representations, and select the best matching candidate from among the tens of thousands of words in the reader’s vocabulary. Contemporary research on reading and visual word recognition seeks to better understand these coding, matching and selection processes. A critical question regarding orthographic input coding concerns the nature of the fundamental units that allow access to lexical information. Do these units represent individual letters, or larger letter clusters, such as bigrams? Furthermore, the obvious follow-up question would be, how is order information then represented across the encoded units?

With respect to this second question, one might wonder whether accurately encoding order information really is critical for visual word identification. Consider, for example, the notorious viral “Cmabrigde Uinervtisy email”:

*(1) It deosn’t mttaer in waht oredr the ltteers in a wrod are*, *the olny iprmoatnt tihng is taht the frist and lsat ltteer be at the rghit pclae*.

Most readers appear to find sentence (1) relatively easy to understand, despite the fact that most of its words are misspelled due to a reordering of their letters. On the face of it, this observation lends support to the claim embodied within the email, i.e., that preserving the order of the interior letters of a word is not necessary for successful visual word recognition.

A possible response to this claim would be:

*(2) Is the cgdirbmae eiaml a ccerrot doitpircsen of the pnemacidert fecad by rredaes wehn the cartnel prat of the snirtg is resreved*? ^1^

Sentence (2) seems somewhat harder to read than sentence (1) (Is the cambridge email a correct description of the predicament faced by readers when the central part of the string is reversed?), even though it conforms to the two criteria specified in the Cmabrigde Uinervtisy email, in that a) the exterior letters of each word are preserved, and b) all of the interior letters are present, but in a different order. Why is there such a difference between sentences (1) and (2)?

The difference may be due to the specific form of jumbling used in sentence (2), in which the interior part of the word has been reversed (e.g., “string” became “snirtg”). However, an additional factor may also be at work here. That is, one of the things that may make it difficult to recognise the intended word in examples like *pnemacidert*, *cartnel* and *resreved* is that these examples trigger partial recognition of other words (e.g., *pneumatic*, *cartel* and *reserved*), and that the activation of these words interferes with recognition of the intended word. In some contemporary theories of word recognition, such interference effects would be a direct consequence of the mechanisms underlying normal reading, whereas other theories have no obvious means to account for such effects.

The present paper offers an investigation of the issues raised by these anecdotal observations, with a specific focus on the effect of letter string reversal. We used computational modelling to derive theoretical predictions and masked form priming methodologies to test these predictions experimentally. In the following, we begin by introducing the masked form priming methodology, before turning to theoretical predictions concerning the effect of letter string interior reversal. Although careful consideration of the assumptions of different theoretical accounts allows one to understand why string interior reversal might disrupt word identification, generating experimental predictions requires one to run simulations of computational models. We report such simulations (and describe how other researchers can reproduce these simulations) and use these predictions to motivate a series of three experiments. The results of these experiments are consistent with one of the existing computational models—the Spatial-coding model [1]—but pose a critical challenge to other current models. As discussed below, contemporary models of visual word recognition do agree that this manipulation of letter order should be particularly disruptive, but, as will be discussed below when those models are presented in some detail, the models disagree with respect to just how disruptive, and why.

## Masked form priming

Much of our extant knowledge about orthographic input coding has been amassed in experiments using the masked priming paradigm [2]. In a conventional masked priming experiment, a briefly presented (e.g., 50 ms) lower case prime is preceded by a forward mask (e.g., ######) and is followed by an upper case target which serves as a backward mask. Typically, a lexical decision response is required to the target. When the prime is a nonword that is orthographically similar to the target (e.g., *anster*-ANSWER), lexical decision latencies are shorter than when the target is preceded by a dissimilar nonword. This phenomenon is referred to as “form priming”.

Critically, form priming effects are observed even when the letters shared by the prime and target occur in different positions in the two letter strings. The initial demonstrations of such priming effects involved relatively subtle misorderings, such as transpositions of adjacent letters (e.g., *gadren-GARDEN*) [2–5] or letters separated by a single intervening letter (e.g., *caniso-CASINO*) [6]. In these transposed letter priming experiments, primes with two transposed letters (e.g., *jugde*-JUDGE) produced larger priming effects than orthographic control primes involving two substituted letters (e.g., *jupte*-JUDGE). Subsequent work [7] investigated more extreme transpositions, establishing that significant priming could be obtained when using primes in which several pairs of adjacent letters are transposed (e.g., *sdiwelak-SIDEWALK* and *isdewakl-SIDEWALK*), although not when using primes in which all of the successive pairs of letters are transposed (e.g., *isedawkl-SIDEWALK*; see [8] for a replication).

The focus of the present research is the effect of reversing the interior letters of a word (“RI” manipulations, such as *c**etupmo**r*-COMPUTER). The impact of RI primes was initially examined in a masked form priming experiment [9] in which the critical related primes were formed by reversing the interior letters of 7-letter target words (e.g., *cartnel-CENTRAL*). Control (“unrelated”) primes were formed by replacing the interior letters with different letters while maintaining the end letters (e.g., *civfdyl-CENTRAL*). The results showed only a nonsignificant 2 ms difference between these conditions. Whitney et al. ([9], p. 113) concluded that, “This pattern of results is clearly in favor of the predictions of OB [open bigram] models and against the predictions of Spatial-coding. The OB coding scheme correctly predicts this pattern because it explicitly encodes letter order”. To better understand this conclusion we next describe the different theoretical assumptions associated with these models, focusing on their predictions concerning RI primes.

### What makes reversed string pairs sufficiently dissimilar so that they produce no priming?

#### Explanation 1. Letters in reversed strings are not in the correct (absolute) position

According to early models, the standard approach to questions concerning the nature of the fundamental units that allow access to lexical information and the way in which order information is represented across those units was to assume that words are activated by position-specific letter units. That is, it was assumed that there are separate channels (or “slots”) for each possible letter position. For example, the word *cat* would be coded by activating the three letter codes *C*_1_, *A*_2_, and *T*_3_, whereas the word *act* would be coded as *A*_1_, *C*_2_, and *T*_3_ (where the subscript indexes letter position). This approach was adopted by the most well-known computational models of visual word recognition and naming [10–15].

This position-specific (i.e., slot) coding approach offers a straightforward explanation of why a reversed interior string like *cetupmor* is perceptually dissimilar to its base word *COMPUTER*. Other than the exterior letters, each of the letters is in a different position in the two strings; that is, the interior letters of *cetupmor* activate the “wrong” letter units for *COMPUTER*. (We use an 8-letter example in our subsequent discussion as well as in our experiments because it makes the comparison cleaner—in a 7-letter word reversing the interior letters leaves the middle letter in the same position.) Thus, a prime like *cetupmor* is no more similar to *COMPUTER* than a control prime such as *cifagnar*.

Although it has the virtue of simplicity, the slot coding approach does not explain the phenomenon illustrated by the original Cmabrigde Uinervtisy email, i.e., the relative ease with which jumbled words can sometimes be read *wehn tehir ltteers are not in the rghit pclae*. Moreover, this approach has now been falsified by a wealth of empirical evidence, including the masked form priming evidence discussed above. For example, transposed letter (TL) priming effects are problematic for the slot coding approach because both TL primes (e.g., *jugde-JUDGE*) and control replacement letter primes (e.g., *jupte-JUDGE*) mismatch the target word at exactly two letter positions. Therefore, according to this approach, they should be equally similar to their target, implying that they should not differ in their effectiveness as primes. This and other problems with the slot coding approach [16] have led to several proposals for alternative orthographic input coding schemes, as reviewed below (see [17–18] for more extensive reviews of these and other relevant data).

#### Explanation 2. String reversal disrupts the order of most of the letter pairs

One class of current models explains perceptual similarity effects such as the effects of TL similarity as the direct consequence of the nature of the representational units that are used to encode the relative order of the letters in a word. The general class of such models can be referred to as *local context* models, with the most successful instances being open-bigram models. Word identification in these models is based not on individual letters, but on letter pairs (including noncontiguous letter pairs) called open bigrams (OBs). For example, the set of OBs required to code the word *judge* includes the contiguous letter pairs *JU*, *UD*, *DG*, *GE* as well as the noncontiguous letter pairs *JD*, *JG*, *UG*, *UE*, and *DE*. A standard assumption is that there are some constraints on the number of OBs activated, with the main constraint in the most cited OB model [4] being that OBs are not activated for pairs of letters that are separated by more than two intervening letters. (The motivation for this constraint follows from the data reported in [4].) Transposing adjacent letters has only a minimal effect on the set of open bigrams activated when reading a word/nonword. For example, exactly the same set of open bigrams is required to represent the word *judge* and the transposed letter version *jugde*, with the single exception that the bigram set for the latter stimulus includes *GD* (rather than *DG*). Hence, these models have no difficulty explaining the large priming effects produced by TL primes [2–6].

According to local context models, string reversal, on the other hand, should completely disrupt the set of open bigrams in the target word; for example, *cat* and *tac* share no open bigrams (*CA*, *CT*, *AT* vs *TA*, *TC*, *AC*). That is, according to this approach, *cat* and *tac* are no more similar than an all-letter-different pair like *cat* and *dov*. Likewise, RI primes and targets share no open bigram units in the version of OB coding proposed by [4]. In another version of open-bigram coding [19], RI primes and targets do share two bigrams—the “edge” bigrams that code the initial and final letters along with their adjoining spaces—but these are also shared by the orthographic control primes and their targets in both [9] and the present experiments. Therefore, according to these OB models, the prime *cetupmor* is no more similar to the target word *COMPUTER* than is the prime *cifagnar*. The finding of no priming for RI primes [9] is therefore consistent with these models. (There is a version of OB coding, called overlap OB coding, which combines elements of both the local context and position overlap approaches. We delay discussion of this scheme until the General Discussion, so as to focus on the basic distinction between position overlap models and local context models).

#### Explanation 3. Letters in reversed strings are not close enough to their correct positions

Another class of current models explains perceptual similarity effects such as TL similarity effects as the direct consequence of uncertainty regarding the positions of the transposed letters. Such models include the Spatial-coding model [1], the Overlap model [20], the LTRS model [21] and the Bayesian Reader model [22–23]. This uncertainty implies that there is some overlap in the position representations of adjacent letters; for this reason, we refer to this class of models (i.e., letter-based models that incorporate letter position uncertainty) as *position overlap* models.

**Fig 1:**
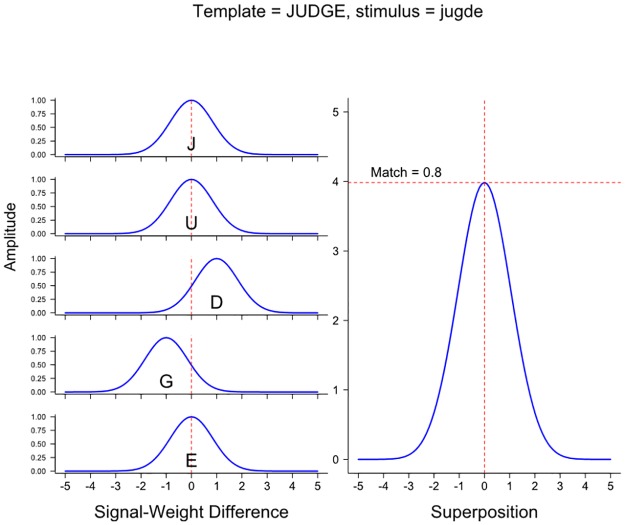
Match computations in the Spatial-coding model at the JUDGE word node when the input stimulus is the transposed-letter nonword *jugde*.

Position overlap models include one or more parameters to represent the extent of letter position uncertainty. That is, the values of these parameters represent the likelihood that the letters *D* and *G* in *jugde* occur in their appropriate order (GD) rather than in their transposed order (DG). How the effect of letter position uncertainty is represented in the Spatial-coding model is illustrated in Fig 1. On the left-hand side of the Figure one finds the functions that are computed by the *judge* word node when *jugde* is presented. These functions represent the difference between a noisy position signal from each letter and a weight that corresponds to the expected position of each letter in a particular word (in this case, *judge*). More specifically, as shown on the left-hand side of the Figure, separate signal-weight difference functions are computed for each of the letters in the reference word. In the case of the letters *J*, *U*, and *E*, the difference functions are centered on a value of 0, because these letters occur in corresponding positions in *judge* and *jugde*. The difference function for the letter *D* is centered on +1, because this letter occurs one position later in the stimulus *jugde* than in the reference word *judge*. The difference function for the letter *G* is centred on -1, because this letter occurs one position earlier in the stimulus *jugde* than in the reference word *judge*.

The “match value” of the presented letter string (*jugde*) and the word *judge* (i.e., their orthographic similarity) is calculated based on the graph shown on the right-hand side of Fig 1. The graph represents the superposition function (i.e., a normalized combination of all the difference functions seen on the left-hand side of the Figure). The maximum value of the superposition function (see the horizontal line) is the match value. (The Spatial-coding model also includes two inputs that reflect the match between the exterior letters of the reference word and the input stimulus; these are not shown in Fig 1.)

As can be seen in Fig 1, the difference functions all have a certain width (which depends on a parameter *sigma*) and that width is selected to ensure that there is some overlap between the functions for the transposed letters and the functions for the correctly positioned letters. The existence of this overlap means that the peak of the superposition function (which, as noted, determines the match value for the two letter strings according to the model) is increased by the contribution of the difference functions created by the transposed letters. Thus, to the extent that the functions for the transposed letters overlap the 0-based difference functions created by the correctly positioned letters, a TL letter string results in a better match value with the reference word (i.e., a higher peak) than does a double substitution letter string (e.g., *jupte*). If the functions were quite narrow (i.e., if the assumption was that there was relatively little position uncertainty) the transposed letters would not contribute to the superposition function created by the difference functions for the correctly positioned letters and, hence, to the final match value. Thus, the model would not predict a TL priming effect (i.e., setting the *sigma* parameter to zero would eliminate the model’s ability to explain TL priming effects—see [1] for a demonstration of this point with respect to the Spatial-coding model).

**Fig 2:**
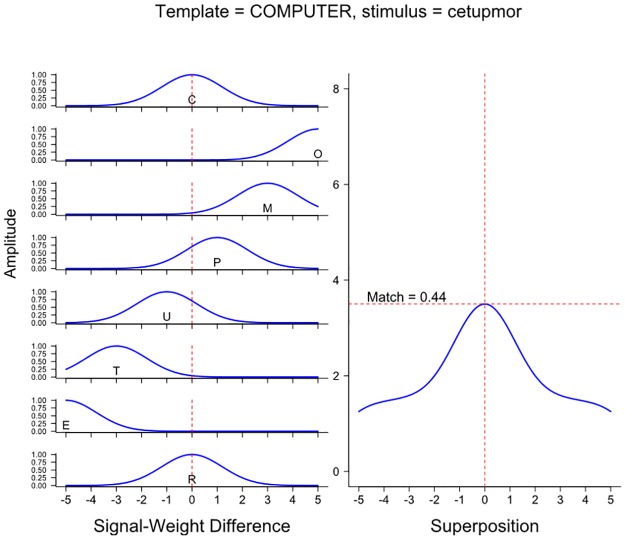
Match computations in the Spatial-coding model at the COMPUTER word node when the input stimulus is the reversed-interior prime *cetupmor*.

**Fig 3:**
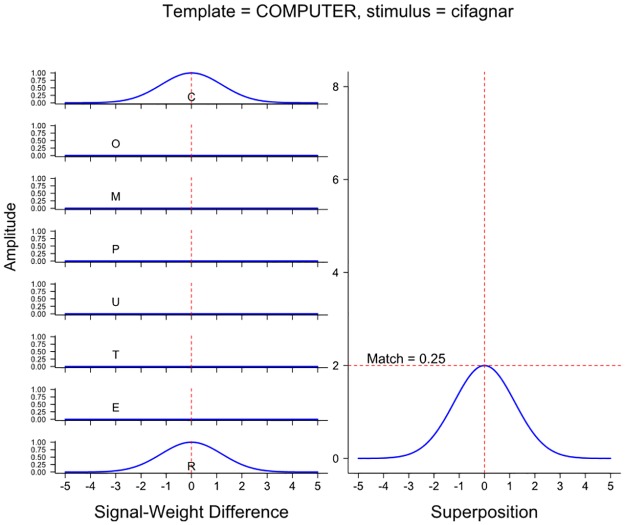
Match computations in the Spatial-coding model at the COMPUTER word node when the input stimulus is the relevant unrelated prime *cifagnar*.

With respect to the specific comparison investigated in the present experiments (RI primes such as *cetupmor* for COMPUTER), according to the Spatial-coding model, those RI primes are moderately similar to their targets, because the exterior letters are in the correct position and the middle letters of the RI prime are reasonably close to the positions they occupy in the target (e.g., the *U* and *P* of *cetupmor* are both only one letter position away from the positions they occupy in the target *COMPUTER*). This similarity is illustrated in Fig 2, which shows the components of the match computation in the Spatial-coding model. (For comparison purposes, an identical calculation involving the corresponding unrelated RI prime, *cifagnar*, and COMPUTER is shown in Fig 3.) Nonetheless, as can also be seen, despite containing the same letters, *cetupmor* and *computer* are only moderately similar, because half of the letters in *cetupmor* are somewhat far from the positions in which they are occur in the word *computer*.

#### Summary of different theoretical explanations of reversed-string similarity

To summarise the information presented thus far, the three broad theoretical accounts reviewed above differ with respect to how letter position is coded, leading to different predictions about how disruptive letter string reversal should be and why. Position-specific (slot) coding and local context (e.g., OB) models agree that reversed string pairs like *cetupmor* and *computer* should be no more similar to each other than control pairs like *cifagnar* and *computer*, but they disagree as to why. According to position-specific coding, string reversal is disruptive because it results in letters being in the incorrect absolute positions. According to local context coding, string reversal is disruptive because it disrupts the order of most of the letter pairs in the string. By contrast with these two accounts, position overlap models allow for uncertainty regarding the relative position of the letters in a string—indeed, this uncertainty is the basis of TL similarity in these models. These models can tolerate letters not occurring in their correct absolute position; what is critical is how far the letters are from their correct position (where “correct position” is defined in absolute terms in the Overlap model and in more dynamic terms in the Spatial-coding model). In the case of RI pairs like *cetupmor* and *computer*, several letters are too far from their expected positions to constitute a good match, but the exterior letters and the medial letters are well placed and do contribute to a match. Consequently, position overlap models predict that RI primes are moderately similar to their targets. The string reversal manipulation is therefore of theoretical interest because it clearly differentiates the theoretical match values predicted by position overlap and local context models.

#### Deriving predictions from models

Although the different theoretical models predict different match values for reversed string pairs, it need not follow that the models make different predictions about the outcome of masked form priming using these pairs. The dissociation between match values and masked form priming effects has been demonstrated in our previous work (e.g., [1,8,24]). For example, Guerrera and Forster’s [7] T-All primes (e.g., *ocpmture*-COMPUTER) are also moderately similar to their targets according to the Spatial-coding model (match = 0.5), but simulations of the model show that it predicts (correctly) that there will be no priming for these primes. Thus, to establish what a computational model such as the Spatial-coding model actually predicts for RI primes it is necessary to run simulations.

We ran simulations of the masked form priming version of the lexical decision task using the *easyNet* software package [25]. We simulated three different models: a position-specific coding model (the interactive-activation model [13]), the Spatial-coding model [1], and an open-bigram coding model (the relative position model [26], referred to as the RPM-IA). The same 8-letter word vocabulary was used for each of the models. For the simulations of the interactive activation and RPM-IA models a simple threshold of 0.68 on word level activity was used to trigger *Yes* responses; the Spatial-coding model incorporates a model of lexical decision, and this model was used to make lexical decisions using the same parameters as in [1] (including a lexical identification threshold of 0.68 on word level activity). Further details of all of the simulations can be found in Appendix A with the simulation and experimental stimuli being reported in Appendix B.

In order to provide additional points of contrast, the behavior of two other models was also simulated: the Bayesian Reader model [22–23] and the LTRS model [21]. These models were simulated using source code/applications provided by the first authors of the respective papers. Although the Bayesian Reader and LTRS models were not originally the focus of our investigation, both models make predictions about masked form priming effects in the lexical decision task, and it therefore seemed relevant to evaluate their predictions. Before discussing the results of the simulations we first briefly describe these two models.

#### Bayesian Reader

The Bayesian Reader model [22–23] is based on the idea that the input from each letter in the word being read is noisy both with respect to the letter’s identity and its location. Initially, therefore, the system only has a vague idea of both letter identities and letter positions. Over time, however, the information about both aspects becomes less noisy allowing the system to more accurately identify those aspects. As that information becomes less noisy, what is being continually updated is the support the acquired information provides for the various possible responses that are relevant to the task at hand. Specifically, in a lexical decision task, this information provides support for either a word or a nonword response. (In contrast, in normal reading the information would provide support for the various possibilities for the word being read.) When the support is sufficient (e.g., the calculated probability becomes, for example, .95 that the letter string is a word), a lexical decision response will be made.

Form priming from orthographically similar primes (including transposed letter primes) arises because processing the prime has already provided some support for the target. Therefore, the amount of evidence needed from target processing for the “word” threshold to be reached is reduced. Essentially, the evidence produced by the two stimuli is merged in coming to a conclusion about the correct response. As Norris and Kinoshita [23] have shown, this model provides a good account of a number of word recognition phenomena.

#### The LTRS model

Like the Bayesian Reader model, the LTRS model [21] also assumes that the nature of letter perception is dynamic and stochastic. Letters’ identities and their orderings are discovered over time as the noisy input is clarified. However, unlike the Bayesian Reader model, negative evidence is regarded as being completely diagnostic. More specifically, as information about the presented letter string is identified, word units consistent with that information are activated. However, as soon as any evidence is identified that rules out a particular word (e.g., as soon as the system realizes that the G and D are not in the correct order for the word *judge* when the prime *jugde* has been presented), the on-going (i.e., prime-driven) activation of the relevant word units stops, allowing that unit’s activation to decay. The amount of priming that a given prime will produce, therefore, will be a function of how long the word unit has been activated by that prime which will be a function of how long it will take for the system to discover a piece of negative information.

This model can, of course, readily explain why primes with letter substitutions produce less priming than identity primes; because priming ceases as soon as a letter that is inconsistent with the target becomes available, the more letters that differ between the prime and the target, the sooner, on average, that target activation based on the prime will terminate. It also can explain why transposed-letter primes (e.g., jugde-JUDGE) are more effective than replacement-letter primes (e.g., jupte-JUDGE). The former type of prime does not become inconsistent with the target until the identities of both of the transposed letters have become available and their relative positions have become apparent, whereas the replacement-letter primes become inconsistent with the target as soon as the identity of either of the replacement letters becomes available. Adelman [21] showed that the LTRS model provides a very good fit to a broad range of form priming phenomena.

#### Simulation results

The results of the model simulations are shown in Table 1 (see column headed “Standard Masked Priming”). Not surprisingly, the IA and RPM-IA models predict no priming for reversed-interior primes. In both cases, this is because the reversed-interior prime is no better a match for the target than is the unrelated prime, although the underlying assumptions about the orthographic representations vary between the models, as noted above. More surprisingly, perhaps, the simulation of the Spatial-coding model shows that this model also predicts no priming for reversed-interior primes, despite the fact that these primes result in noticeably greater match values than the unrelated primes in this model. That is, in the Spatial-coding model, these primes, which are only moderately similar to their targets, are unable to support priming in a conventional masked priming lexical decision task.

**Table 1:** Standard and sandwich priming predictions for five different computational models.

	Standard Priming			Sandwich Priming		
Model	Related	Unrelated	*Effect*	Related	Unrelated	*Effect*
**IA**	16	16	*0*	16	16	*0*
**RPM**	25	25	*0*	28	28	*0*
**LTRS**			*13*			
**Bayesian Reader**	456	508	*52*			
**SCM**	151	154	*3*	160	180	*20*

Note: The LTRS model makes predictions regarding the magnitude of priming effects, but not the mean time taken to perform lexical decision for individual conditions. Sandwich priming cannot be simulated in the current versions of the LTRS and Bayesian Reader models.

The other two models that we simulated do predict priming for reversed-interior primes (see Table 1). Indeed, the Bayesian Reader model predicts substantial priming (52 ms) in the conventional masked priming lexical decision task used in Experiment 1. This model does assume a reasonably similar orthographic input coding scheme to that assumed by the Spatial-coding model, although the details of the matching algorithms clearly differ. Therefore, it produces a higher similarity score for the RI primes than for the unrelated primes used here. However, where the two models differ substantially is with respect to lexical selection mechanisms and the ways in which the process of lexical decision making is implemented. It is these differences that produce the substantially different predictions concerning the impact of moderately similar primes. The LTRS model also predicts a priming effect in Experiment 1, however, the prediction is for a somewhat smaller effect (13 ms) than that predicted by the Bayesian Reader model.

In summary, the position-specific, local context and Spatial-coding models predict negligible or no RI priming, whereas the Bayesian Reader model predicts substantial facilitory priming and the LTRS model predicts moderate facilitory priming. It is worth emphasising again that different models can make similar predictions for different reasons (i.e., IA, RPM and Spatial-coding model predict no/negligible priming for different reasons) and, as well, different models can make different predictions even though they make fairly similar assumptions about orthographic coding (i.e., the Spatial-coding, Bayesian Reader and LTRS models). The goal of Experiment 1 was to test these predictions.

## Experiment 1

### Method

#### Participants

The 24 participants were undergraduates from the University of Western Ontario who received course credit for their participation. All reported having normal or corrected-to-normal vision. All three experiments were approved by the office of Human Research Ethics at the University of Western Ontario. Participants in all three experiments provided informed written consent before participating.

#### Stimuli and apparatus

The target stimuli were 48 eight-letter words and 48 orthographically legal eight-letter nonwords. The mean frequency of the target words was 27.6 per million (range = 1–165) [27]. The mean neighborhood size (calculated using the N-Watch software [28]) was 0.2. There were no repeated letters in any of the word targets. The related primes for both target types were constructed by using the first and eighth letter of the target and reversing the order of the second through the seventh letters (e.g., the related prime for the target COMPUTER was *cetupmor*). The unrelated primes for both target types were orthographically legal nonwords which began and ended with the same letters as the target (e.g., the unrelated prime for COMPUTER was *cifagnar*). As noted, the primes and targets are listed in Appendix B.

The experiment was run using DMDX experimental software [29]. Stimuli were presented on a SyncMaster monitor (Model No. 753DF). Presentation was controlled by an IBM-clone Intel Pentium. Stimuli appeared as black characters on a white background. Responses to stimuli were made by pressing one of two <shift> keys on the keyboard.

Each participant saw each target only once. To successfully counterbalance the stimuli, both the word targets and the nonword targets were arbitrarily divided into two sets of size 24. One set of each type of target was primed by a related prime, with the other set being primed by an unrelated prime for half the participants. For the other half of the participants, the sets of both word and nonword targets were primed by the opposite prime type.

#### Procedure

Participants were run individually. Each participant sat approximately 18 inches in front of the computer screen. Participants were instructed to respond to strings of letters presented on the computer screen by pressing the right <shift> key if the letters spelled an English word or the left <shift> key if the letters did not spell a word. They were also told that a string of number signs (i.e., “########”) would appear prior to the string of letters. They were not told of the existence of the prime, but were asked to respond to each target as quickly and as accurately as possible.

Experiment 1 followed a standard masked priming methodology. On each trial the participants were presented with the string of number signs for 550 ms. The prime was then presented in lower case for 55 ms. The target then appeared in upper case for either three seconds or until the participant responded. Participants performed eight practice trials before beginning the experiment and were given the opportunity both during the practice trials and immediately afterwards to ask the experimenter any questions in order to clarify any confusion concerning what was required.

### General analysis procedure

The same procedure was used to analyse the data in each of the experiments reported here. Error trials and trials with latencies longer than 1500 ms were removed from the latency analyses. Tests showed that doing so never resulted in the exclusion of significantly more trials from either of the two prime conditions. Items or participants with error rates greater than 25% were excluded from the analyses (no participants were excluded for this reason). Two (Prime Type) x 2 (List) ANOVAs were performed with both subjects and items as random factors. List is a between-subject and between-item factor which was included as a dummy factor in order to remove variance due to the counterbalancing of stimuli across conditions [30]. These ANOVAs were performed separately for each response category (words and nonwords in Experiments 1 and 2; “same” and “different” trials in Experiment 3).

### Results

The percentage of trials removed due to latencies exceeding 1500 ms was 0.1% for the word trials and 2.2% for the nonword trials. The mean latencies and error rates in Experiment 1 are shown in Table 2. Target words preceded by RI primes were responded to 4 ms faster than targets preceded by orthographic control primes, a difference that was not significant in either the subjects (*p* = .492) or the items analysis (*p* = .769; both *F*s < 1.0). The error rate was slightly higher in the RI prime condition than in the control condition, a difference that was not significant in the subject ANOVA (*F*_*1*_(1, 22) = 1.64, MSe = .001, *p* = .214) but was marginal in the item ANOVA (*F*_*2*_(1, 46) = 3.83, MSe = .001, *p* = .093). For nonword targets there was no effect of prime type in either the latency or error data (all *F*s < 1.00).

**Table 2:** Mean RT (ms) and error rates (in parentheses) for Experiments 1, 2 and 3.

	Words/same trials			Nonnwords/different trials		
Experiment	Related	Unrelated	*Effect*	Related	Unrelated	*Effect*
**1 (Standard priming)**	619 (2.8%)	623 (1.6%)	*4 (-1%)*	724 (4.8%)	725 (5.8%)	*1 (1%)*
**2 (Sandwich priming)**	643 (3.7%)	666 (3.8%)	*23 (0%)*	753 (4.5%)	735 (3.1%)	*-18 (-1%)*
**3 (Same-different task)**	455 (4.4%)	484 (7.2%)	*29 (3%)*	526 (4.6%)	518 (3.0%)	*-8 (-2%)*

Note: Left hand columns represent word trials for Experiments 1 and 2, and “Same” trials for Experiment 3. Right hand columns represent nonword trials for Experiments 1 and 2, and “Different” trials for Experiment 3.

### Discussion

The results of Experiment 1, using a conventional masked priming methodology, showed a null effect of RI primes. This pattern of results is problematic for the Bayesian Reader model, which predicts strong priming for the RI primes used in this experiment. It is also problematic for the LTRS model, although that conclusion is tempered by the facts that a) the model did not predict a large effect and b) numerically, there was a small (4 ms) priming effect. However, these results are consistent with the other theoretical accounts discussed previously, though for quite different reasons. Slot-coding models predict an absence of priming due to the fact that the targets share exactly the same number of letters (in the same positions) with the RI and unrelated primes. That is, those types of models regard the RI and unrelated primes as equally similar to the target. Local context coding models predict an absence of priming because the reversal of the target’s letters in the RI primes disrupts local context in a way that makes the RI primes and their targets perceptually dissimilar. By contrast, the RI primes and their targets do have reasonably similar orthographic input codes according to the Spatial-coding model. That model, however, also predicts that no priming should be obtained, due to the dissociation between match values and priming effects in the standard masked priming paradigm. The goal of Experiment 2 was to obtain evidence that might adjudicate between the successful accounts of the data from Experiment 1.

## Experiment 2

### Sandwich priming

In addition to demonstrating the dissociation between match values and form priming effects in the conventional masked priming paradigm when the primes and targets are moderately similar, Lupker and Davis [8] presented a new masked priming technique for overcoming this dissociation. In this technique, the target itself is initially presented as a masked prime *on both related and unrelated prime trials*. It is then followed by the prime of interest, either related or unrelated and then by the target. In essence, the prime of interest is sandwiched between the two presentations of the target, hence, the name, “sandwich priming”. The intended impact of presenting the target itself as an initial prime is to give the activation of the target’s lexical representation a boost. This boost may counteract lexical inhibition that the target node receives from its competitors. Alternatively, it may be that the pre-activation of the target can be maintained by a somewhat similar prime but not by an unrelated prime (the second possibility is actually a somewhat more important factor in terms of how the sandwich priming technique enhances priming effects in the implemented Spatial-coding model).

Lupker and Davis [8] demonstrated the success of the sandwich priming technique by examining priming effects for Guerrera and Forster’s [7] T-All (ocmutpre-COMPUTER) primes. Simulations of the Spatial-coding model predicted the statistically null effect that Guerrera and Forster produced in the conventional masked priming task as well as predicting that priming should be obtained if the sandwich priming technique were used. The results of a sandwich priming experiment confirmed this prediction, showing a T-All priming effect of 40 ms (Lupker and Davis also replicated Guerrera and Forster’s statistically null effect in the conventional masked priming task). The finding of a strong priming effect for T-All primes when sandwich priming is used is consistent with the Spatial-coding model.

### Using sandwich priming to adjudicate between local context and Spatial-coding models

To summarize the argument so far, we have seen that the local context and Spatial-coding models can make similar predictions, but also differ in a number of ways, ways that should be able to lead the models to make distinct predictions. The TL priming effect is predicted by both models, despite the fact that the explanation of the effect differs noticeably. RI primes, however, do have the potential to allow us to distinguish between these two types of models, because there is a qualitative difference in the predictions that the models make regarding the similarity between RI primes and their targets. Local context models predict no similarity between RI primes and their targets (i.e., a match value of zero). It follows that local context models predict no RI priming whether conventional or sandwich priming is used. By contrast, the Spatial-coding model (and other position overlap models) do predict some similarity between these primes and targets. Nevertheless because this similarity is relatively modest it is necessary to conduct simulations of the full model in order to determine the exact predictions made by the models in a sandwich priming experiment.

The results of simulations of the Spatial-coding model for the RI primes are shown in Table 1. As noted, the model predicts a negligible priming effect (of 3 cycles) for RI primes when the conventional priming technique is used, consistent with the results of Experiment 1. In the simulation of sandwich priming, however, the Spatial-coding model predicts a 20 cycle priming effect. The position-specific models, in contrast, predict no priming in either priming paradigm because both the RI primes and the unrelated primes match their targets in exactly two positions (i.e., the initial and final letter positions).

The aim of Experiment 2 was to test the above predictions. According to local context models, no priming should be observed for RI primes. By contrast, the Spatial-coding model predicts a reasonable size priming effect when the sandwich priming technique is used. (One would expect that the Bayesian Reader and LTRS models would also predict facilitory priming in this situation given that they predict priming in the conventional task, however, the software we used to simulate these models does not allow one to simulate the sandwich priming methodology, and thus we are unable to directly confirm this expectation.)

### Method

#### Participants

The 32 participants were drawn from the same population as in Experiment 1. All reported having normal or corrected-to-normal vision.

#### Stimuli & design

The experimental design and the prime and target stimuli were the same as in Experiment 1.

#### Procedure

The procedure in Experiment 2 was identical to that of Experiment 1 except that the number signs were followed by the presentation of the target in lower case letters for 33 ms, prior to the prime of interest.

### Results

The percentage of trials removed due to latencies exceeding 1500 ms was 0.8% for the word trials and 3.4% for the nonword trials. The mean latencies and error rates in Experiment 2 are shown in Table 2. For word targets, the latency data showed a 23 ms advantage for related primes which was significant in both the subject (*F*_*1*_(1, 30) = 9.83, MSe = 864.93, *p* < .005) and item analyses (*F*_*2*_(*1*, *46)* = 12.95, MSe = 1049.24, *p* < .005). There was no effect of prime type in the error data (both *F*s < 1.0). For nonword targets, there was a marginal effect of prime type in the latency data with RI primes showing an 18 ms disadvantage over control primes (*F*_*1*_(1, 30) = 3.84, MSe = 1373.29, *p* = .059; *F*_*2*_(1, 46) = 2.97, MSe = 2626.71, *p* = .092). Error rates to nonwords were also slightly higher in the RI condition than in the control condition, however, this difference was not significant (both *p*s > .10).

### Discussion

The results of Experiment 2 are consistent with the predictions of the Spatial-coding model, as shown in Table 1. Although the model (correctly) predicts essentially no RI priming when using the conventional masked priming methodology, it predicts a reasonable RI priming effect when using the sandwich priming methodology, the exact pattern we observed. According to the model, RI primes produce a moderately high match value with their target words (because these primes overlap with the target both at the ends and, to some degree, in the middle), and the use of sandwich priming enables this moderate match value to be manifested as a facilitory priming effect.

In contrast, the results of Experiment 2 are problematic for local context models. According to these models, either the RI primes and targets share no bigram units or (in the case of the SERIOL model) those bigrams that are shared by RI primes and targets are also shared by the unrelated primes and targets used here (i.e., the “edge” bigrams that code the initial and final letters along with their adjoining spaces). Either of these situations should result in no priming effect.

In summary, the results of Experiment 2 are quite consistent with the predictions of the Spatial-coding model [1] and (presumably) the current Bayesian Reader model (Norris & Kinoshita, 2012) and the LTRS model [21], but are problematic for local context models such as the discrete open-bigram models [4,26], the LCD model [31], and the SERIOL model [19] as well as for position-specific models [10–15]. These results also provide further evidence of the utility of the sandwich priming methodology in enabling priming to be obtained in situations where it is not obtained using the more conventional masked form priming methodology [8].

## Experiment 3

The significant priming from RI primes in the sandwich priming task used in Experiment 2 provides good support for position overlap models. RI primes match their targets sufficiently well to be able to produce clear priming effects, at least when the target is pre-activated. The sandwich priming technique is not, however, the only experimental technique that allows an examination of orthographic priming effects when the target is pre-activated. Norris and colleagues [22, 32–33] have presented good evidence that their sequential presentation masked prime same-different task accomplishes much the same goal.

In this task, an initial stimulus (the reference) is presented for approximately 1 s. It is followed by the brief (e.g., 50 ms) presentation of a masked prime and then by the presentation of a target stimulus. The task is to indicate whether the reference and target are the same or different. On “same” trials, primes that are orthographically similar to the reference/target facilitate responding, with the priming effect being essentially the same size regardless of whether the reference/target stimulus is a nonword, a low frequency word or a high frequency word [22,32]. Further, the task appears to be immune to morphological priming effects [34–35].

On “different” trials (when the reference and the target do not match), related trials can involve primes that are related to either the reference or the target. When they are related to the target, no priming [22,32]. When they are related to the reference, however, there is an inhibitory priming effect [33, 36–38], presumably due to the fact that the orthography of the prime supports an incorrect “same” response. What, therefore, appears to be crucial in this experimental paradigm is the orthographic relationship between the prime and the reference stimulus rather than the prime and the target.

This pattern of results, particularly the fact that there typically are large priming effects for nonwords, have led Kinoshita and Norris [32] to propose that “the same-different task holds considerable promise as a tool for examining the nature of prelexical orthographic representations. The task appears to tap into the same representations that support word recognition but not to be influenced by the lexical retrieval processes” (p. 13), and “This evolving prelexical orthographic representation is both input to the lexical access process and the representation used in the same-different task” (p. 14). If these claims are correct, the task does provide a second means of evaluating the question of whether RI primes are orthographically similar to their targets/reference stimuli, as claimed by the position overlap models, or whether they are not similar, as claimed by the local context models. Specifically, the former models would predict RI priming effects in this task while the latter models would not. Experiment 3 was an examination of these predictions. Note that in Experiment 3, the related primes on “different” trials were related to the reference stimuli and involved the same RI relationships that we used for related primes on “same” trials. Therefore, if the position overlap models are correct, an additional prediction would be for inhibitory RI priming effects on “different” trials.

### Method

#### Participants

There were 60 participants in Experiment 3, drawn from the same population as in Experiments 1 and 2. All reported having normal or corrected-to-normal vision.

#### Stimuli and apparatus

For the “same” trials the prime and reference/target stimuli were the stimuli used on the word trials in Experiments 1 and 2. To create the “different” trials in Experiment 3, 96 eight-letter words were selected, that were not used on the “same” trials. These words were then divided in half, with one half of the words to be used as reference stimuli and the other half of the words to be used as targets. The related primes on “different” trials involved the same RI relationship between the reference and the prime that we used for related primes on “same” trials. The stimuli on “different” trials are listed in Appendix C.

The experiment was run using DMDX experimental software [29] in a multi-use lab. Stimuli were presented using IBM-clone Pentium computers on one of three monitors (a SyncMaster (Model No. 753DF), a Dell (Model No M782p) or an ADi MicroScan (Model LM-1764)). As in previous experiments, the stimuli appeared as black characters on a white background. In all other ways, the stimulus presentation processes and the counterbalancing procedures were identical to those in Experiments 1 and 2.

#### Procedure

Participants were run in small groups using individual computers, monitors and keyboards for each person. Each participant sat approximately 18 inches in front of their computer screen. On each trial, initially a reference word was presented in lowercase for 1000 ms above a forward mask “#######”. The reference stimulus and mask then disappeared with the mask being replaced by the prime which remained on the screen for 50 ms. The uppercase target then appeared in the same position as the prime and remained on the screen until the participant responded.

Participants were asked to decide whether the reference and the target were the same word. Participants were instructed to respond by pressing the right <shift> key if the two words were the same or the left <shift> key if they were different. They were not told of the existence of the prime. The trials were presented in a different random order for each participant. Prior to the experimental session, participants received 4 practice trials. The practice stimuli were chosen according to the same criteria as used in the experimental trials and contained triplets not found in the experimental lists.

### Results

The analyses and trial removal criteria were the same as in Experiments 1 and 2. The percentage of trials removed due to latencies exceeding 1500 ms was 0.3% for the “same” trials and 0.4% for the “different trials. The mean latencies and error rates in Experiment 3 are shown in Table 2. On same trials, the mean correct latency for target words preceded by RI primes was 29 ms faster than for targets preceded by unrelated primes. This difference was significant in both analyses (*F*_*1*_(1, 58) = 50.72, MSe = 481.86, *p* < .001; *F*_*2*_(1, 46) = 59.54, MSe = 373.31, *p* < .001). There was also an effect of prime type in the error data (*F*_*1*_(1, 58) = 11.24, MSe = 0.002, *p* = .001; *F*_*2*_(1, 46) = 14.10, MSe = 0.001, *p* < .001) due to there being a lower error rate in the RI condition.

On “different” trials, the mean correct latency for targets when the prime involved a RI transformation of the reference was 8 ms slower than when the target and reference were unrelated. This difference was marginal in both analyses (*F*_*1*_(1, 58) = 3.11, MSe = 718.15, *p* = .083; *F*_*2*_(1, 46) = 3.48, MSe = 471.97, *p* = .069). There was, however, a significant prime type effect in the error data (*F*_*1*_(1, 58) = 4.73, MSe = 0.002, *p* = .034; *F*_*2*_(1, 46) = 7.42, MSe = 0.001, *p* = .009) as the error rate was higher in the RI condition.

### Discussion

The results of Experiment 3 are consistent with the predictions of the position overlap models. Those models claim that an RI transformation produces a letter string that is orthographically similar to the untransformed string/word. Consequently, priming should result when a sufficiently sensitive methodology is used. Further, working under the assumption that, on “different” trials, primes orthographically similar to the reference stimuli will make responding more difficult, these models predict inhibitory effects from RI primes on those trials. This pattern is exactly what was observed. On “same” trials, RI primes produced a 29 ms priming effect in the latency data and a 2.8% priming effect in the error data. On “different” trials, RI primes produced a marginal 8 ms inhibition effect in the latency data and a significant 1.6% inhibition effect in the error data.

By contrast, the results of Experiment 3 are once again problematic for local context models. Most of these models predict that RI primes either share no bigram units with their targets/reference stimuli or (in the case of the SERIOL model) that those bigrams that are shared by RI primes and the targets/reference stimuli are also shared by the unrelated primes and the targets/references stimuli used here. Either of these situations should result in no priming effect, contrary to what was observed.

## General discussion

The aim of the present research was to evaluate models of the orthographic input coding and lexical selection processes that are critical for visual word recognition. The experiments reported here used three different variants of the masked priming paradigm with the main goal being to test between two classes of orthographic input coding models (local context models and position overlap models), as well as among three types of position overlap models (as represented by the Spatial-coding model, the Bayesian Reader model and the LTRS model). We begin by discussing the distinction between local context and position overlap models, before turning to the implications of our results for the position overlap models.

### Local-context (i.e., open-bigram) vs position overlap models

In general, local context and position overlap models tend to produce quite similar match values between a given prime and target and, hence, make very similar predictions concerning masked form priming in many circumstances. However, one aspect of the local context models that does allow them to be differentiated from position overlap models is their assumption that there is no position uncertainty in the way that letters are coded. That is, the letter sequence XY activates the open bigram XY but not the open bigram YX. This aspect of the models allowed us to test between the two model types by using primes that, while sharing all their letters with their targets, shared no more open bigrams with their targets than those same targets shared with their unrelated primes. Specifically, we used primes that reversed the six interior letters in their targets (e.g., *cetupmor*-COMPUTER).

According to almost all of the current local context models, these primes share no open bigrams with their targets. According to those models, then, these primes should provide no priming for their targets in any situation, a prediction that was supported in the conventional masking priming lexical decision task (Experiment 1). According to position overlap models (e.g., the Spatial-coding model, the Bayesian Reader model, the LTRS model, and, although we have not discussed it, the Overlap model), however, there is a moderately high orthographic match between these primes and their targets. Therefore, although at least some of those models also predict a null effect in Experiment 1, the models further predict that those primes and targets should produce priming in situations where the main determinant of form priming is the orthographic match between the prime and the target (i.e., the sandwich priming and masked prime same-different task paradigms).

More specifically, a key feature of Experiment 2 was the use of the sandwich priming technique with the goal being to pre-activate the target such that it would be more susceptible to priming by a moderately similar prime. Simulations of a specific computational model (the Spatial-coding model [1]) led us to predict that the primes of interest would not lead to significant priming when the conventional masked priming technique was used (i.e., Experiment 1), but that priming would be obtained when the sandwich priming technique was used. The results of Experiment 2 are completely consistent with that prediction. It is not possible to conduct simulations of masked priming with the Overlap model [20], so we will not make any claims regarding the implications of our results for that model, other than to note that the model’s predicted match values are at least broadly consistent with those that would be required to produce the results observed in Experiment 2. The same cannot be said of the local context models we considered in the Introduction, however, namely the discrete open-bigram model [4] and the SERIOL model [19]. Our results, therefore, provide a critical challenge to those models.

A similar argument follows from the results of Experiment 3. Priming effects in the masked prime same-different task appear to be mainly driven by the orthographic similarity of the prime and the reference stimulus [22, 32–33]. Therefore, the existence of priming in Experiment 3 implies that the reversed-interior transformation produces letter strings at least somewhat similar to those of their reference stimuli. This type of effect is clearly consistent with the position overlap models. However, it creates a considerable challenge for the open-bigram models, according to which RI primes either share no open bigrams with their reference stimuli or whatever orthographic similarity there is between the reference stimuli and their RI primes also exists between the reference stimuli and their unrelated primes.

One version of the open-bigram model that may be able to account for the present data a little better is the overlap open bigram (OOB) model [39]. This model includes the assumption that letter strings activate transposed-letter bigram units; for example, the stimulus *cat* weakly activates the open bigrams *ac* and *ta*. In this respect, the OOB model incorporates aspects of both the local context and position overlap approaches. In principle, the activation of reversed order bigrams may help the OOB model explain priming for RI primes. For example, the prime *cetupmor* will activate the transposed letter bigrams *ec*, *te*, *ut*, *pu*, *mp*, *om*, and *ro*. Five of these seven bigrams are, of course, also contained in COMPUTER and, crucially, they are not contained in the unrelated prime *cifagnar*. To the extent that these bigrams play an important role in the orthographic coding process, a model of this sort would have some potential to explain the present results.

In practice, however, it is not clear that the OOB model could capture the present priming effects. Grainger et al. [39] suggested that the activation level of the transposed letter bigram units should, of necessity, be quite low; otherwise, the model would encounter difficulty in carrying out basic word identification. Given the parameter settings proposed by Grainger et al. [39], the match between RI primes and their targets/reference stimuli is only 0.25. It is not clear that a match value of this magnitude could support facilitory priming effects of the size we observed in Experiments 2 and 3. However, because we have not simulated an OOB model, we cannot rule out the possibility that a version of the OOB model that implemented lexical decision and masked priming could be made consistent with the present results.

As an alternative approach, one could, of course, attempt to account for the data patterns in Experiments 2 and 3 in other ways, ways that would, potentially, not be inconsistent with the open-bigram position. With respect to Experiment 2, for example, one could argue that the initial prime is presented too briefly to allow the establishment of a full orthographic code. For example, perhaps that code only contains the first four letters of that prime which, in the sandwich priming paradigm, are also the first four letters of the target. (The assumption being made is that processing is serial, however, this assumption is not required.) When the prime of interest is next presented, the active orthographic code maintains the letters that have already been established and adds the letters of the second prime in the positions that were left empty. For example, when the sequence is computer-cetupmor-COMPUTER, the initial prime establishes the code comp???? and the second prime turns it into the code comppmor. The result would be an activated orthographic code that does contain some relevant open bigrams (specifically, co, cm, cp, om, op and mp) which could facilitate target processing (we would like to thank Carol Whitney for suggesting this idea). While this possibility cannot be excluded (as we do recognize that the exact mechanisms underlying sandwich priming are not fully understood), the challenges facing this type of account would not be minor. The first issue that would need to be dealt with is why the second prime wouldn’t simply overwrite the letters that had been established by the initial prime. The general assumption in virtually all models of orthographic coding/letter perception is that in masked priming situations the presentation of a new set of letters means that any ongoing activation of an initial set of letters is stopped. There would be no obvious reason why something similar would not happen here.

A second issue is whether a prime sharing only a few open bigrams units with its target (6 in the present example, with both the prime of interest and the target activating 18 open-bigram units due to them both being eight letters long), would be sufficient to produce a priming effect of the size observed here. That is, the question would be, are, for example, comppmor and COMPUTER sufficiently similar to produce a 23 ms priming effect? As demonstrated in [8] primes typically completely lose their ability to prime when they mismatch in more than two positions. Therefore, it seems unlikely that this level of overlap (i.e., 6/18 open bigrams) could produce the effect observed in Experiment 2.

An alternative way to explain the data from Experiment 2 which would not be inconsistent with the open-bigram position would be to invoke the idea that there is some priming occurring as a result of letter, rather than open-bigram, activation. That is, the assumption is that the first level activated when viewing a letter string is the letter level. Under normal circumstances, it is those representations job to activate the open-bigram representations which are responsible for producing most of the observed priming as well as completing the word recognition process. What can be assumed, however, is that the letter level representations also directly activate word representations for words containing those letters. Therefore, both the initial prime (e.g., computer) and the RI prime (cetupmor) would both activate the target COMPUTER due to the fact that they share letters with COMPUTER, producing priming in comparison to when the prime of interest is cifagnar.

As with the alternative account discussed above, this account must also deal with the problem of explaining how merely activating the eight letters in the target without providing any order information (which can only come from activating open bigrams) could produce a 23 ms priming effect. Those same eight letters would also be activated by RI primes in the conventional task and there is little evidence that a manipulation of that sort produces priming (Experiment 1). Indeed, there are a number of examples of primes that contain all the letters of their targets but in a different order, for example, T-All primes, ocpmture-COMPUTER [7–8] and primes that transpose the first four and last four letters, pmocretu-COMPUTER [7] providing virtually no priming in a conventional task. Thus, it’s quite unclear how the letter to word level linkages alone could have been strong enough to have produced the amount of priming observed here.

Indeed, a similar idea has been proposed in response to data from the same-different task [40–41]. The idea here is that matching in that task is performed largely on the basis of individual letters, rather than at the open-bigram level. Once again, though, such an approach raises the question of, if open bigrams units play no role in the same-different task, what role they play in normal reading?

With respect to the same-different reported in Experiment 3, the equations provided in Whitney [40] lead to the prediction that our RI primes have a same-different task-relevant match value with their targets of .57, while the unrelated primes, because they contain the same first and last letters as the reference stimuli, have a match value of .25. Therefore, the RI primes should produce a priming effect that is 32% of the priming available from repetition primes in this task. This prediction would be in line with the observed priming effect if the expected repetition priming effect in the same-different task was taken to be on the order of 90 ms. This latter value is broadly consistent with the magnitude of repetition priming effects in that task that have been observed using shorter words [22, 32–33]. Thus, this idea could explain the basic phenomenon of the existence of priming for RI primes in Experiment 3 (though not, of course, for the existence of priming in lexical decision tasks such as that in Experiment 2 or in Experiment 2 in [42]).

What does seem clear is that, in order to remain viable, open-bigram models must allow for the activation/impact of transposed letter bigrams in some fashion. Thus, our results impose further constraints on the set of plausible open-bigram models. This outcome continues the theoretical development resulting from previous masked priming experiments [4,16]. These previous experiments have ruled out versions of open-bigram coding in which bigram activation is a function of serial position [43], or in which open-bigram units are independent of serial position but are activated irrespective of the number of intervening letters [26].

### Implications of our results for the Bayesian Reader Model

The Bayesian Reader has no means of explaining the absence of priming for form primes that are moderately similar to their targets. Indeed, the Bayesian Reader model predicts strong facilitory priming not only for RI primes but also for T-All primes and Reversed-Halves (*pmocretu*-COMPUTER) primes. Each of these predictions is incorrect, as has now been demonstrated in several experiments (Experiment 3 in [8], [9], and Experiment 1 of the present paper).

The development of the Bayesian Reader model over its various iterations [22–23, 33] has seen considerable modifications to the nature of its orthographic input coding scheme in response to new data. For example, the slot coding scheme used in the original implementation of the model would not capture transposed letter priming effects (e.g, [3–6]) and, of course it would also fail to capture the facilitory priming effects we observed in Experiments 2 and 3. In contrast, the coding scheme adopted in the current instantiation of the model is much closer in spirit to that in the Spatial-coding model (whereas the Spatial-coding model incorporates a superposition matching algorithm, matching in the current Bayesian Reader model involves a calculation of Levenshtein distance), although the models’ approaches to matching letter strings differ. This theoretical development of the model is a positive aspect of its underlying framework, and the responsiveness of the modelling to new data provides a nice illustration of the proper cycle of model construction, testing and refinement. Nevertheless, as Bowers and Davis noted [44], the adaptiveness of the model to handling challenging data undermines the claim that the critical aspects of the Bayesian Reader are driven entirely by rational analysis or fundamental Bayesian principles. Two important questions for future research are a) whether the model can capture data from our experiments, and b) if, as we suspect, explaining these data requires a modification to the model’s current approach to lexical selection, whether the modified model should still be considered a “Bayesian” Reader.

### The LTRS model

Although our experiments focused on two competing classes of orthographic coding models, it should be noted that a somewhat different type of approach, although it can be characterized as a position overlap approach, has been proposed by Adelman [21] in his LTRS model. The LTRS model explains form priming effects in terms of the dynamic and stochastic nature of feature extraction from printed words with the level of priming reflecting the duration during which a prime remains potentially consistent with the target. For example, in this model, primes with letter substitutions produce less priming than identity primes because prime-driven target activation ceases as soon as a letter that is inconsistent with the target becomes available. Hence, the more letters that differ between the prime and the target, the earlier in prime processing the target will be rejected as a potential candidate, on average. Transposed-letter primes (e.g., jugde-JUDGE) are more effective than replacement-letter primes (e.g., jupte-JUDGE) because the former primes do not become inconsistent with the target until the identities of both of the transposed letters have become available and their relative position is discovered, whereas the replacement-letter primes become inconsistent with the target as soon as either of the replacement letters is identified.

Adelman [21] showed that the LTRS model provides a very good fit to a broad range of form priming conditions. We used the program provided by James Adelman to obtain exact priming predictions for our prime conditions in the conventional masked priming task, using the parameters from Adelman [21]. Those predictions, as reported in Table 1 were for priming of 13 ms for the RI prime. Thus, the model overestimated the priming effects observed in Experiment 1. Nevertheless, the prediction is not wildly different from the obtained priming effect (4 ms) and, therefore, it is certainly possible that the model may be able to obtain a better fit to the data in Experiment 1 with a different set of parameters. Predicting the large increase in priming observed in Experiments 2 and 3 would appear to be a somewhat larger challenge without adding new assumptions to the model. The reason is that, in its current formulation, the priming mechanism in the model would act identically in all three paradigms.

In conclusion, the results of the present experiments provide further evidence of the utility of the sandwich priming methodology, as well as the masked prime same-different task methodology, in enabling priming effects to be examined in situations where they are not obtained due to complicating factors intrinsic to the more conventional masked form priming methodology [8]. We argue that this feature of these methodologies makes them more sensitive techniques with which to test the sometimes subtle differences in predictions made by different contemporary orthographic coding models. In the case of the experiments reported here, these methodologies made it possible to detect evidence of the similarity of reversed-interior letter strings to their forward versions. This evidence is consistent with the predictions of the Spatial-coding model [8], but poses a critical challenge to open-bigram models, models in which letter position is coded in terms of ordered letter pairs.

## Appendix A

### Methodology for running simulations of the models

#### Vocabulary

The same vocabulary was used in simulations of each of the models (except for the LTRS model, which does not simulate lexical processing, and hence does not make use of a vocabulary). This vocabulary consisted of all of the 8-letter words contained in the SUBTLEX_UK corpus [45] with Zipf scores of 2 or more and no non-alphabetic characters; the resulting vocabulary contained 8895 words. Resting activities for the IA, RPM-IA and SCM models were derived from log (SUBTLEX_UK) word frequency using the same formula as in the original IA model (see [46], p. 216).

The easyNet software was used to run simulations of the IA, RPM-IA and SCM models. The easyNet software, scripts and associated files required to reproduce these simulations can be downloaded via this website: http://adelmanlab.org/easyNet/downloads/.

#### SCM simulations

Masked priming and lexical decision were simulated in the same way as in [1]. The prime durations in cycles were identical to the prime durations in ms used in Experiments 1 and 2, i.e, 55 cycles for the prime and (in Experiment 2) 33 cycles for the initial prime. The model parameters were identical to the default values specified in [1], with the exception that the top-down connection from words to letters was omitted. In practice, top-down feedback in the model is only relevant to cases where the prime is a subset of the target, and this modification has no effect on the simulation results here (see [47] for discussion of the implementation of top-down feedback in the model and its consequences for subset primes). However, switching off top-down feedback enables simulations of the model to run considerably faster, as this allows a shortcut in which match values are computed only once per stimulus field per trial.

#### IA simulations

IA simulations were conducted using the easyNet implementation of the original IA model. To simulate the latency of Yes responses in the lexical decision task, an absolute threshold was applied to the words layer; the threshold was set to 0.68, i.e., a Yes response was made as soon as any word node exceeded this value (there was a single target stimulus—*clothing*—for which this threshold was not attained). In this model, durations in cycles can be multiplied by 10 to convert them to a scale comparable to milliseconds, and thus the prime duration was set to 5 cycles (3 cycles for the initial prime in the sandwich priming experiment).

#### RPM-IA simulations

The procedure for running RPM-IA simulations was identical to that for IA simulations, with the exception that the model selected was “Relative Position” rather than “IA”. We used a PD of 5 cycles.

#### LTRS simulations

Predictions from the LTRS model were obtained by running the executable provided by James Adelman at his website: http://www.adelmanlab.org/ltrs/ (a simplified version of LTRS can be simulated using the current easyNet software, but at present it is necessary to use Adelman’s [21] program to obtain predictions from the full model).

#### Bayesian Reader simulations

To simulate the Bayesian Reader model (the version described in [23]) we compiled the code provided by Dennis Norris [https://www.mrc-cbu.cam.ac.uk/people/dennis.norris/personal/NoisyChannel/] to create an executable program that runs on the (Ubuntu) Linux platform. The executable, scripts and stimulus files required to run the simulations of the Bayesian Reader model reported in this paper can be found here: http://www.bristol.ac.uk/expsych/research/processes/clc/resources/supplementary-materials/davislupkersubmitted/. Because the Bayesian Reader is a stochastic model, each prime-target trial was simulated with 25 separate runs. The same 8-letter word vocabulary used to test the other models was used in simulations of this model.

## Appendix B

(Primes and targets for word and nonword trials in Experiments 1 and 2; the word targets and their primes were also used for “same” trials in Experiment 3)

Related Prime/Unrelated Prime/Word Target
  atulosbe/adefiche/ABSOLUTE
  arosivdy/amacunty/ADVISORY
  athgimly/afdposby/ALMIGHTY
  aotsecnr/arhmivwr/ANCESTOR
  bpurknat/bgomlcit/BANKRUPT
  bnotirae/bvehusie/BARITONE
  bradnuoy/bmitseiy/BOUNDARY
  ceniralt/comusokt/CLARINET
  cnihtolg/cradfukg/CLOTHING
  cetupmor/cifagnar/COMPUTER
  cedisnor/catumvur/CONSIDER
  coitaern/cuyluysn/CREATION
  craobpud/cniudped/CUPBOARD
  dethguar/dobtpior/DAUGHTER
  dhgilyat/dfqukpet/DAYLIGHT
  duasonir/dyecemer/DINOSAUR
  devocsir/dawunmar/DISCOVER
  ditsemoc/dulnaruc/DOMESTIC
  fgnimalo/fpcenuto/FLAMINGO
  fsiruolh/fnecaebh/FLOURISH
  gufetarl/gohidisl/GRATEFUL
  hmosdnae/hcuvtrie/HANDSOME
  irtsudny/icbmakwy/INDUSTRY
  iamrofnl/ievcudsl/INFORMAL
  jsuolaey/jnivtiry/JEALOUSY
  mitsejac/molnupuc/MAJESTIC
  mnahcret/msofvzit/MERCHANT
  mhcranoy/mlswixey/MONARCHY
  mhcatsue/mdnofrie/MUSTACHE
  oatneirl/ouksouml/ORIENTAL
  oedistur/oafomlar/OUTSIDER
  plbatroe/pdfihsue/PORTABLE
  pufrewol/patcinal/POWERFUL
  puoicers/payamaws/PRECIOUS
  psahcrue/pnifmwoe/PURCHASE
  roitcaen/ryulmuyn/REACTION
  ritnamoc/ruksevuc/ROMANTIC
  slbmarce/stfwonxe/SCRAMBLE
  sgatrohe/sqilmude/SHORTAGE
  sedluohr/sakbiafr/SHOULDER
  srehtuon/smildian/SOUTHERN
  setnilpr/sodcabgr/SPLINTER
  slknirpe/stfcampe/SPRINKLE
  smordnye/scawtvpe/SYNDROME
  tanimrel/tovuscol/TERMINAL
  tnasuohd/tmecield/THOUSAND
  tlgnaire/tkpsoume/TRIANGLE
  vacitrel/vomuknul/VERTICAL
Related Prime/Unrelated Prime/Nonword Target
  ahcnumle/adsrixte/ALMUNCHE
  blutsrip/bhakmcop/BIRSTULP
  curhtilp/caskfebp/CLITHRUP
  cehpmorn/calgvusn/CROMPHEN
  cehtuorn/cilfaisn/CROUTHEN
  clipsurt/cbagnomt/CRUSPILT
  drahcnet/dmolswit/DENCHART
  dehcnarm/dilsvowm/DRANCHEM
  dokcnirm/dufvsawm/DRINCKOM
  dseknort/dcalvimt/DRONKEST
  felpmard/fitgcond/FRAMPLED
  fihcnarx/folmsewx/FRANCHIX
  gnowhsad/grimkcod/GASHWOND
  gahcnirl/gokmsuwl/GRINCHAL
  hsogapet/hnujiqut/HEPAGOST
  hsrednit/hmcolwut/HINDERST
  khplurae/ktgdisoe/KARULPHE
  lstnugie/lmkrapoe/LIGUNTSE
  lanehtir/lomubfor/LITHENAR
  muqraloe/mipsetie/MOLARQUE
  mrehtlon/mcudfkin/MOLTHERN
  pnetrald/pcofsokd/PLARTEND
  pignuolt/paqrieft/PLOUNGIT
  pnekcard/pmotsiwd/PRACKEND
  pnelward/pmibsock/PRAWLEND
  petsdorm/pilnkawm/PRODSTEM
  rsnulcah/rwmotvih/RACLUNSH
  rsehcnut/rmilvwat/RUNCHEST
  snemract/swovzuxt/SCARMENT
  sepmulct/sigrafnt/SCLUMPET
  sgdnirce/splzawme/SCRINDGE
  smedirct/snolavzt/SCRUDENT
  senruohl/sacmiafl/SHOURNEL
  sanruoht/simcielt/SHOURNAT
  sopmurhn/sagcewln/SHRUMPON
  senagilt/sumopukt/SLIGANET
  staprulh/sbegcidh/SLURPATH
  sridlomt/scafbint/SMOLDIRT
  sraklipt/smofhegt/SPILKART
  sedralpn/sobcukgn/SPLARDEN
  shguarpt/stjoenqt/SPRAUGHT
  setfirpm/sokbungm/SPRIFTEM
  sednirpl/sofmacgl/SPRINDEL
  samuorpt/sineicgt/SPROUMAT
  seliauqy/sotoyopy/SQUAILEY
  tdocsnae/tlivmzue/TANSCODE
  tshguare/tnlpiome/TRAUGHSE
  wetsmiar/wodncour/WAIMSTER

## Appendix C

(Reference stimuli, primes and targets on “different” trials in Experiment 3)

Reference/Related Prime/Unrelated Prime/Target
  ushering/unirehsg/ucamokvg/FRACTION
  hospital/hatipsol/hufeqrel/PROCLAIM
  butchery/brehctuy/bsalmdey/PHYSIQUE
  auditory/arotiduy/asebeley/MAGNETIC
  solitary/sratiloy/snefuduy/LUKEWARM
  morality/mtilaroy/mfubusey/DOCUMENT
  adoptive/avitpode/awefgabe/VELOCITY
  brownies/beinwors/baumcavs/YOURSELF
  blasting/bnitsalg/bsufcodg/KILOGRAM
  ancestry/artsecny/amfvoxzy/BURGUNDY
  chimneys/cyenmihs/cgarwuds/SYMBOLIC
  wrinkles/welknirs/wahbcovs/PROXIMAL
  watering/wniretag/wcosuhog/REDUCING
  chanting/cnitnahg/cruhmelg/SECONDLY
  brawling/bnilwarg/bsukvomg/CHARMING
  youngest/ysegnuot/yrapmiat/WHISTLED
  watching/wnihctag/wrubvkog/BOARDING
  builders/bredlius/bcatfoas/ALERTING
  behaving/bnivaheg/bmurotog/SHREWDLY
  urgently/ultnegry/ufhsajzy/LAUGHTER
  actively/alevitcy/akonudry/SPORADIC
  virtuous/vuoutris/vaeadnes/MAJORITY
  mystical/macitsyl/morubnjl/SECURITY
  adultery/aretludy/anihkoby/MISTAKEN
  vanquish/vsiuqnah/vneopmoh/CONSUMER
  children/cerdlihn/castbufn/METAPHOR
  boastful/buftsaol/bihdneil/SPECTRUM
  watchmen/wemhctan/wurksfin/MANIFEST
  backfire/brifkcae/bsuldvoe/DISTANCE
  blackout/buokcalt/bielseft/SOMEWHAT
  chipmunk/cnumpihk/crangulk/BACKSIDE
  strangle/slgnarte/shymivfe/MANIFOLD
  weakling/wnilkaeg/wsudboug/SOFTWARE
  tranquil/tiuqnarl/teogsecl/SANDWICH
  brochure/bruhcore/bsifzane/FESTIVAL
  province/pcnivore/pzsuwaxe/JUDGMENT
  champion/coipmahn/ceuqrekn/STRAINED
